# Effects of Plasma ZrN Metallurgy and Shot Peening Duplex Treatment on Fretting Wear and Fretting Fatigue Behavior of Ti6Al4V Alloy

**DOI:** 10.3390/ma9040217

**Published:** 2016-03-23

**Authors:** Jingang Tang, Daoxin Liu, Xiaohua Zhang, Dongxing Du, Shouming Yu

**Affiliations:** 1 Institute of Corrosion and Protection, School of Aeronautics, Northwestern Polytechnical University, Xi’an 710072, China; jingangtang@163.com (J.T.); yhzhangxh@nwpu.edu.cn (X.Z.); ddx@mail.nwpu.edu.cn (D.D.); shoumingyu@163.com (S.Y.); 2 Institute of Machinery Manufacturing Technology, China Academy of Engineering Physics, Mianyang 621900, China

**Keywords:** fretting fatigue, fretting wear, titanium alloy, plasma zirconium nitride, residual stress

## Abstract

A metallurgical zirconium nitride (ZrN) layer was fabricated using glow metallurgy using nitriding with zirconiuming prior treatment of the Ti6Al4V alloy. The microstructure, composition and microhardness of the corresponding layer were studied. The influence of this treatment on fretting wear (FW) and fretting fatigue (FF) behavior of the Ti6Al4V alloy was studied. The composite layer consisted of an 8-μm-thick ZrN compound layer and a 50-μm-thick nitrogen-rich Zr–Ti solid solution layer. The surface microhardness of the composite layer is 1775 HK_0.1_. A gradient in cross-sectional microhardness distribution exists in the layer. The plasma ZrN metallurgical layer improves the FW resistance of the Ti6Al4V alloy, but reduces the base FF resistance. This occurs because the improvement in surface hardness results in lowering of the toughness and increasing in the notch sensitivity. Compared with shot peening treatment, plasma ZrN metallurgy and shot peening composite treatment improves the FW resistance and enhances the FF resistance of the Ti6Al4V alloy. This is attributed to the introduction of a compressive stress field. The combination of toughness, strength, FW resistance and fatigue resistance enhance the FF resistance for titanium alloy.

## 1. Introduction

Fretting wear (FW) is a surface damage phenomenon that results from small oscillating slipping between two contact solid materials. Fretting fatigue (FF) is a phenomenon of reducing the fatigue resistance that results from FW in contact with other materials under cyclic loading. FF damage is the main failure mechanism between compressor blade rabbets and mortises of aircraft engine, fasteners and other similar components. The effect of fretting contact has been reported to reduce fatigue strength by 20% to 70% [1,2]. Titanium alloy has a high strength, good thermal stability and fine corrosion resistance and is therefore used to fabricate compressor blades, disks of aviation engines and fasteners. However, titanium alloys are sensitive to FF damage because of their low hardness, poor thermal conductivity and high friction coefficient. FW and FF damage are areas of “intense research” in aviation and fretting tribology [3,4,5,6]. In general, FF damage is initiated from surface, and surface modification technology is important to strengthen the FF resistance of titanium alloys [7,8,9,10,11,12]. FW and fatigue stress exist simultaneously in FF damage. It is thus difficult to improve FF resistance by improving wear resistance and fatigue strength, because technologies to do so are generally inconsistent. In general, surface modification to increase FW resistance does not inhibit FF failure [13,14]. Research indicates that nitriding and plasma surface metallurgy could enhance the wear resistance of titanium alloy, but is not advantageous to plain fatigue and FF behavior, because the treatment reduces the toughness [15]. Shot peening could inhibit crack propagation by introducing a surface compressive stress, so it is one of the most effective methods to improve plain fatigue and FF resistance [16,17]. However, shot peening alone does not ensure good wear resistance, so does not improve FF resistance effectively especially when FF conditions cause severe wear damage. A combination of wear resistance of the surface metallurgy layer and fatigue resistance of shot peening would solve difficulties of wear and fatigue in FF damage.

Zirconium (Zr) dissolves with α–Ti and β–Ti. In addition, zirconium nitride (ZrN) exhibits a high wear and oxidation resistance [18,19]. Therefore, it is possible that good wear resistance of titanium alloys could be obtained by surface metallurgy treatment following shot peening. Based on the above background, we aimed to fabricate a ZrN metallurgy layer using plasma Zr metallurgy with nitriding on Ti6Al4V (TC4) alloy base. The influence of plasma ZrN metallurgy or shot peening on FW and FF behavior was investigated. This research is aimed to provide a novel technology to improve the FF resistance of titanium alloy parts.

## 2. Materials and Methods

### 2.1. Materials and Samples

Ti6Al4V (6.3% Al, 4.2% V, 0.1% Fe, 0.03% C, 0.015% N, 0.03% H, 0.14% O, balance Ti) was annealed and contained α-Ti and β-Ti phases. The mechanical properties of Ti6Al4V alloy are as follows: 1080 MPa (σ_b_), 1010 MPa (σ_0.2_), 15% (δ), 41% (Ψ).

**Figure 1:**
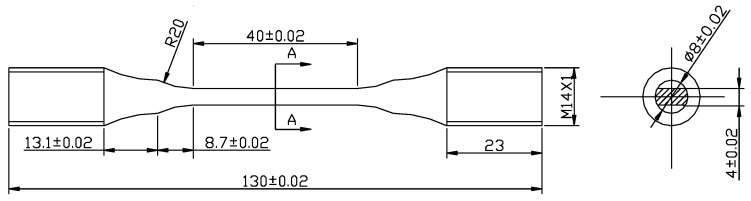
Dimensions and shape of fretting fatigue samples (unit: mm).

The FW samples (30 and 10 mm diameter disks) were polished using SiC abrasive papers from 240# to 1500#. The initial surface roughness *R*a was less than 0.2 μm. The dimensions and shape of the FF samples are shown in Figure 1. The longitudinal surface of the FF samples was polished until the surface roughness *R*a was less than 0.2 μm.

### 2.2. Surface Modification Treatments

**Figure 2:**
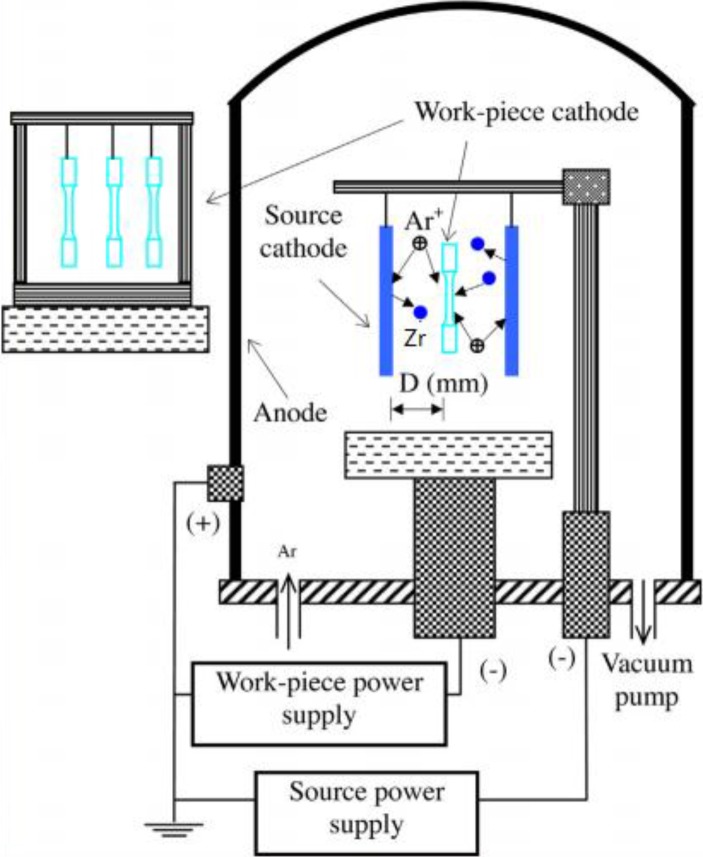
Schematic diagram of glow plasma metallurgy equipment.

Two steps were used to fabricate a ZrN metallurgy layer on Ti6Al4V using self-made glow plasma surface metallurgy equipment. The target was a plate with composition of more than 99.95% Zr. Samples were rinsed in acetone and alcohol using ultrasound to remove grease. (1) The Zr metallurgy treatment principle is shown in Figure 2. The source voltage of the Zr plate was 900 V, the cathode voltage of Ti6Al4V alloy sample was 400 V, the frequency of the pulse power was 10 kHz, the duty ratio was 80% and the processing time was 3 h; (2) Argon and nirogen fluxes of 60 sccm and 75 sccm, respectively, were used for nitriding after the Zr metallurgy step. The chamber pressure was 50 Pa, the process temperature was 850 °C and the processing time was 3 h.

The process parameters for shot peening were as follows. The composition of ceramic shots with an average diameter of 0.30 mm was 68% ZrO_2_ and 30% SiO_2_. The peening intensity was 0.33 mmA (Almen intensity). The ratio of coverage was 100% and the shot blasting angle was 90°.

As-received samples were labeled BM. Samples that were nitrided following plasma surface Zr metallurgy were labeled ZrN. After being shot peened, these samples were labeled ZrN+SP. Shot-peened Ti6Al4V alloy samples were labeled SP.

### 2.3. FW Experiment and FF Experiments

**Figure 3:**
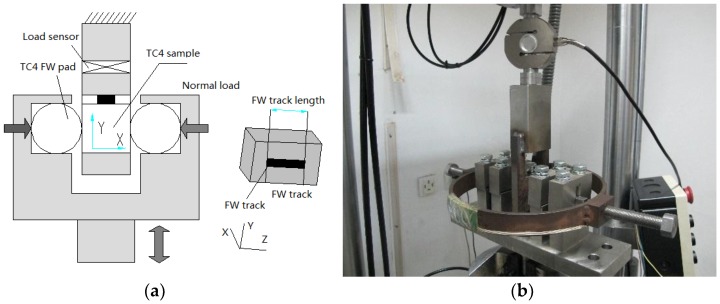
(**a**) Schematic diagram and (**b**) physical device of the fretting wear equipment.

FW experiments and FF experiments were carried out at room temperature. FW experiments were conducted with a 10 kN electrical hydraulic testing machine according to the research [20,21]. An experimental schematic and photograph are shown in Figure 3. The contact mode was by cylinder and plane. The fretting pad was a Ti6Al4V cylinder of 10 mm diameter and 4 mm length. The surface roughness (*R*a) was 0.1 μm and the contact load (*P*) was 50 N/mm. The maximum Hertzian pressure was 354.7 MPa and the initial contact width was 7.1 μm. The oscillating frequency was 5 Hz. The surface friction coefficient was tested using a stress-strain sensor. Testing of the FW volume loss was as follows: contour profile equipment was used to measure the profile of the FW track surface along the oscillating slipping direction, and the cross-sectional area *A* of the FW track was determined by integrating the profile using Origin software (OriginLab Corporation, Northampton, MA, USA). The wear volume loss Δ*V* was obtained from equation Δ*V* = *A*∙*L*, in which *L* is the length of the FW track in the normal oscillating slipping direction.

**Figure 4:**
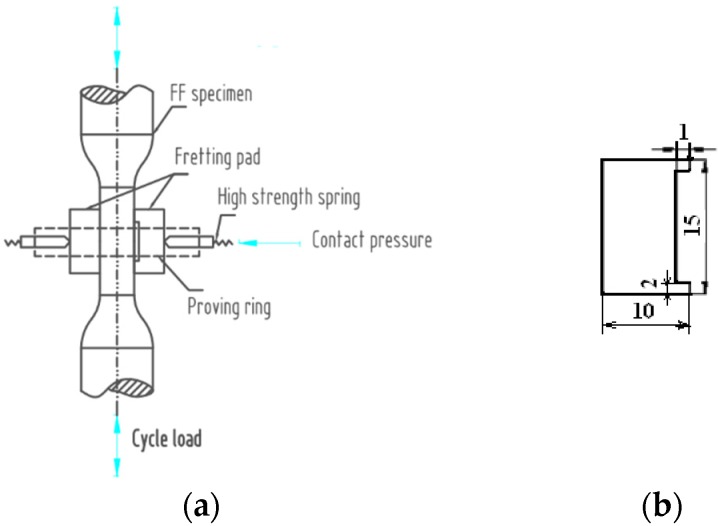
(**a**) Schematic diagram and (**b**) fretting contact pad of FF equipment (unit: mm).

**Figure 5:**
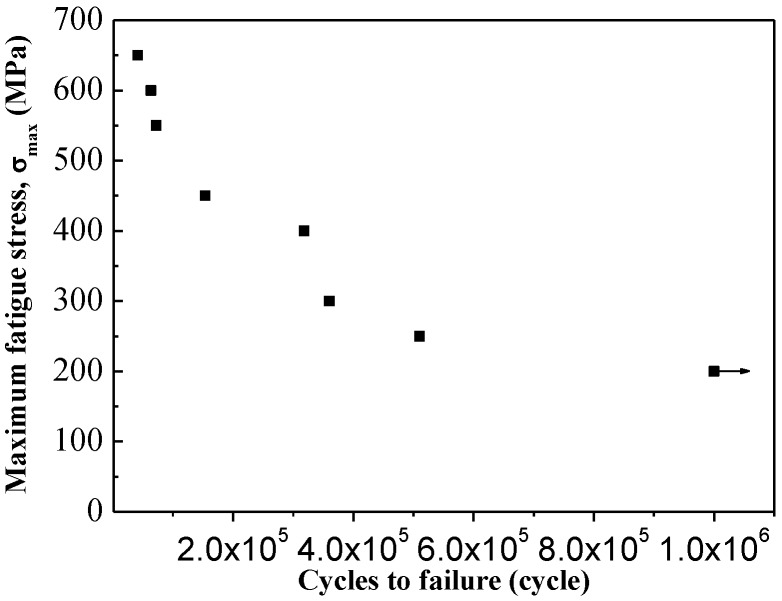
FF life of Ti6Al4V base with maximum fatigue cyclic stress (S-N curve).

FF tests were carried out at a 100 kN GPS 100 fatigue equipment in tensile–tensile loading mode. A schematic diagram of the FF equipment is shown in Figure 4a. A schematic diagram of the fretting contact pad is shown in Figure 4b. A plane–plane contact mode between the fretting pad was used with 2 mm width and 10 mm length. The surface roughness of the FW pads was about 0.1 μm. The contact length between the fretting pad and the specimen was 6 mm. Slipping between the fretting area was realized by elastic strain in the tensile–tensile fatigue process. The amplitude of fretting slipping could be changed by varying the span of the fretting pads. The frequency of the extra sinusoidal fatigue cyclic stress was 110 Hz and the stress ratio was 0.1. Figure 5 shows the FF life of Ti6Al4V base with a maximum fatigue cyclic stress (S-N curve). Extra fatigue maximum stress to investigate the influence of different modifications on the FF life of Ti6Al4V can be determined from Figure 5. The maximum fatigue stresses are 550 and 650 MPa. According to the loading condition of aviation engines, contact stress in the fretting contact area is 85 MPa. The FF life is evaluated from an average of three parallel specimens.

### 2.4. Analysis and Measuring Method

The morphology, phase structure, FW and fracture of ZrN samples were studies using scanning electron microscopy (JEOL Ltd., Tokyo, Japan). Phase patterns were studied by X-ray diffractometry (XRD). The cross-sectional elemental distribution along the depth was determined by glow discharge spectrography. Microhardness was tested using a Knoop microhardness tester in the cross-sectional depth. The pressing head was a Knoop head. The long diagonal line of the pressing track was parallel to the sample surface. The pressing load was 0.98 N, and was maintained for 20 s. The residual stress was studied using an X-ray diffraction stress tester (PANalytical B.V., Almelo, The Netherlands) with Ti target and Ti (110) crystal plane diffraction by the sin2ψ method. The residual stress along the cross-sectional depth was studied by gradual chemical corrosion using a mixture of hydrochloric and hydrofluoric acids. The ductility of the modification layer was evaluated using self-made cyclic pressing testing experiments [21]. The ductility evaluation method was carried out using a Vivtorinox pyramid diamond indenter that was pressed repeatedly onto the sample surface. The pressing head was held in contact with the sample surface throughout. The pressing load was sinusoidal, the stress ratio was 0.1 and the maximum load was 200 N.

## 3. Results

### 3.1. Microstructure and Cross-Sectional Composition

**Figure 6:**
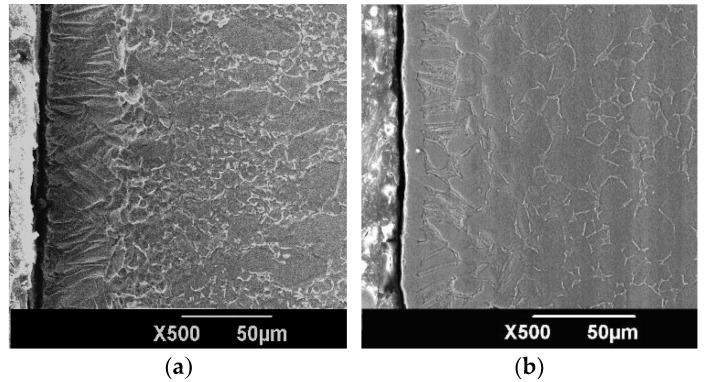
Cross-sectional microstructure of (**a**) ZrN and (**b**) ZrN+SP layers on Ti6Al4V alloy.

**Figure 7:**
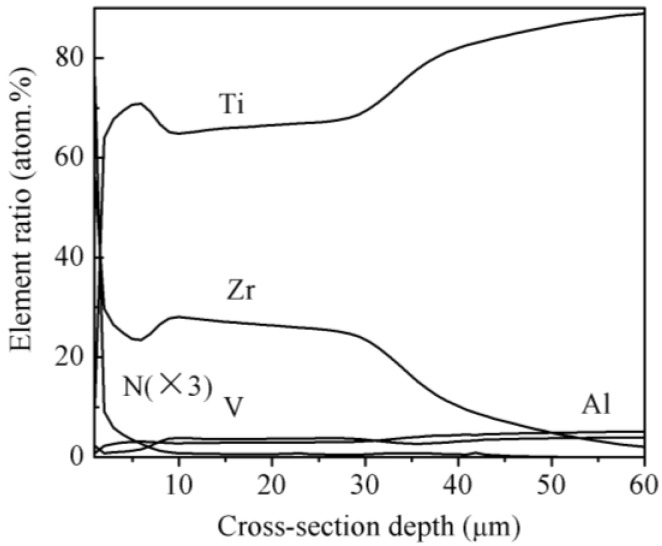
Elemental distribution along depth of ZrN layer on the T6Al4V alloy.

**Figure 8:**
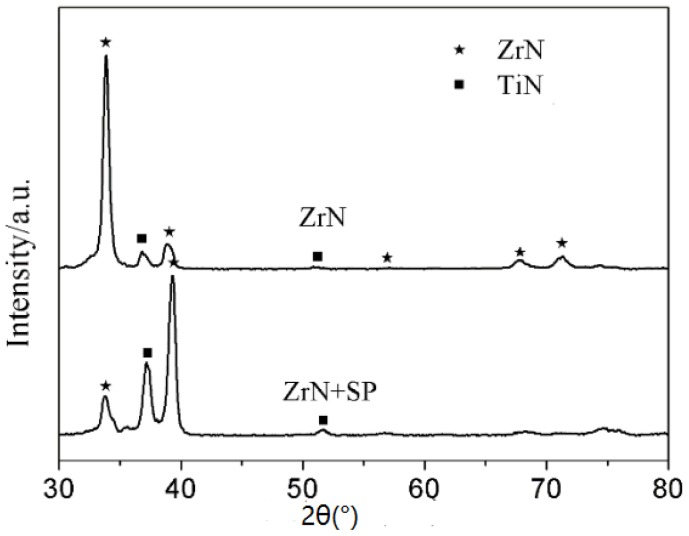
XRD results of the ZrN and ZrN+SP layers.

**Figure 9:**
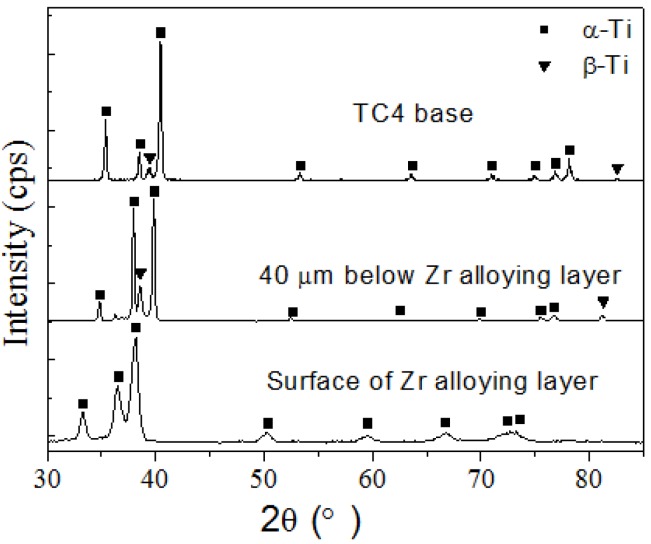
XRD results of plasma Zr alloying specimen.

Figure 6 shows the microstructure morphology of the ZrN layer on Ti6Al4V alloy. Figure 7 shows the cross-sectional elemental distribution of the ZrN layer. Figure 8 shows the XRD analysis results of the ZrN layer. Figure 6, Figure 7 and Figure 8 show that the ZrN layer on Ti6Al4V alloy was ~60 μm thick and that the surface was an ~8-μm-thick nitrogen compound layer, which was composed mainly of ZrN phase and contains little TiN phase. The second layer below the surface compound layer was a 50 μm solution layer with Ti, Zr and few N atoms. Zr and Ti can exhibit a hexagonal close packed (low temperature stable phase: α phase) crystal structure and body centered cubic crystal structure (high temperature stable phase: β phase). Moreover, Zr and Ti had similar atomic number layers after 6 h at 830 °C and a surface layer composition with 40% Ti and 60% Zr. Results indicate that α-Ti and β-Ti existed at 882 °C. The transformation temperature of α-Zr and β-Zr was 865 °C. With increasing Zr solution content in Ti, the above transformation temperature was reduced. Thus, the content of the β phase in the second layer was increased after plasma Zr metallurgy on Ti6Al4V alloy (Figure 9).

Figure 7 shows that the cross-sectional element distribution changs gradually with depth. This indicates that adhesion between the ZrN layer and Ti6Al4V base is metallurgical, which is beneficial for adhesive strength and load-bearing ability. The distribution of elemental N with depth exhibits a significant gradient and low thickness. This occurs because ZrN and TiN are formed with N element diffusion in the Zr layer that hinders diffusion of nitrogen atoms into the sample interior. Thus, the surface compound layer is shallow. A “hollow” existed in the curve of the Zr cross-sectional distribution curve, and “swelling” occurred in the curve of the Ti cross-sectional distribution with depth in the second surface layer of the ZrN layer. This may occur because the adhesive strength between Zr and N is greater than that between Ti and N. This results in the uphill diffusion of Ti from the super-surface to the subsurface.

As shown in Figure 6b, no obvious cracks or delamination existed after post shot peening, and the layer density is improved. XRD results, as shown in Figure 9, indicate that the crystal exhibited a preferred orientation of (111) crystal plane in the ZrN layer. Post shot peening broadened and shifted the Bragg diffraction peak of the ZrN layer to a higher angle. This is attributed to microstructure refining, increasing in dislocation and the introduction of a surface-compressive residual stress in the ZrN layer.

### 3.2. Cross-Sectional Microhardness Distribution

**Figure 10:**
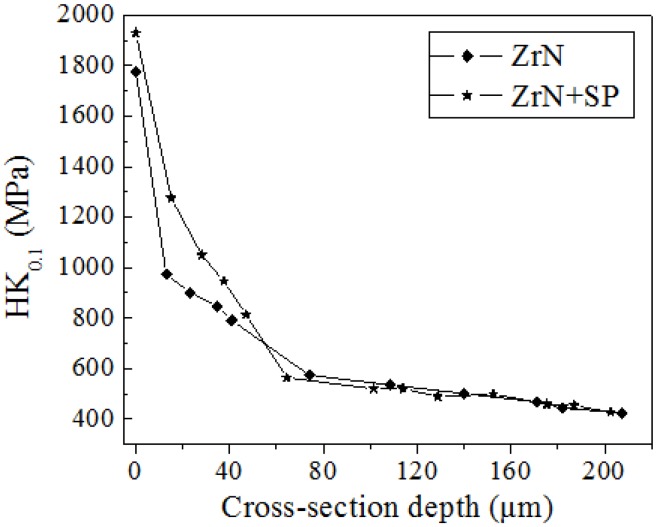
Microhardness distribution along depth of the ZrN and ZrN+SP layers.

Figure 10 shows the cross-sectional microhardness distribution of the ZrN layer. The surface microhardness of the ZrN layer was 1775 HK_0.1_ and 1930 HK_0.1_ after post shot peening, which were both greater than that of Ti6Al4V base (420 HK_0.1_). The microhardness along the depth of both metallurgy layers changed gradually. The gradient change of microhardness with depth is beneficial for improving the load bearing ability.

The high hardness of the ZrN layer on Ti6Al4V is attributed to the formation of ceramic ZrN and TiN because of the formation of interstitial solid–solution with N atom diffusion into the Ti-Zr solid solution. The influence of shot peening on ZrN layer hardness is attributed to the refinement of surface microstructure and work hardening Improving the surface hardness is beneficial for increasing FW resistance of Ti6Al4V alloy.

### 3.3. Compressive Residual Stress Distribution with Depth

**Figure 11:**
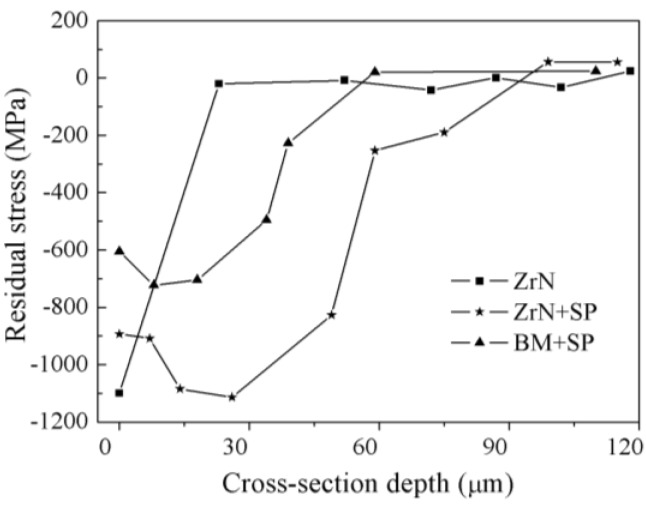
Results of residual stress depth distribution for different state samples.

Figure 11 shows the compressive residual stress distribution profile of ZrN, SP and ZrN+SP sample with depth. The amplitude of the surface compressive residual stress of the ZrN sample was 1000 MPa because of two reasons. Firstly, doping of N atoms with the formation of a ZrN ceramic layer makes the Zr crystal distort after Zr alloying and post nitriding. Secondly, a large divergence exists between the thermal expansion coefficient of ZrN and Ti6Al4V base. The thickness in compressive residual stress field was low and the change gradient was large for the ZrN sample. This type of compressive residual stress field harms fatigue behavior [14]. Shot peening introduces a surface compressive residual stress with amplitude of −604 MPa. The maximum residual stress was −719 MPa. The residual stress field depth was ~60 μm. The introduction of residual stress field is attributed to plastic deformation and strain hardening. The surface residual stress of ZrN samples is attributed to plastic deformation and strain hardening. The surface residual stress of ZrN samples was approximately −897 MPa, which is higher than that of the SP samples alone and lower than that of the ZrN samples alone. Shot peening therefore relaxes the surface residual stress slightly. The residual stress of the ZrN+SP samples was higher than that of the SP samples. This indicates that the Zr–Ti solution layer has a great deformation hardening ability, which is good for improving fatigue resistance.

### 3.4. Ductility of Modification Layer

**Figure 12:**
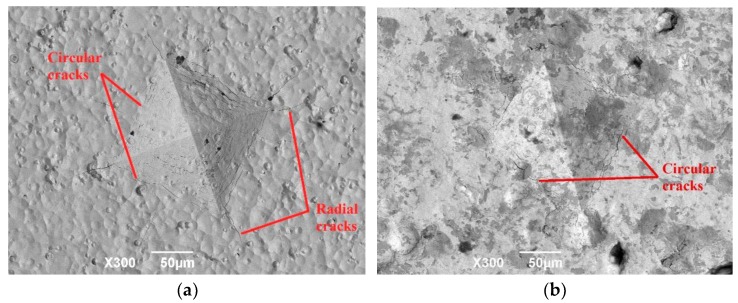
Morphology of track on the (**a**) ZrN and (**b**) ZrN+SP layers.

Ductility of the modification layer is important for its FW and FF behavior. A cycle pressing–pressing indentation method was used to evaluate the apparent ductility after modification. Figure 12 shows the morphology of the indentation track with a 200 N load and 10^4^ cycles. No obvious radial cracks existed with influence of dynamic pressing–pressing loading.

The ductility of the modification layer is important for its FW and FF behavior. A cycle pressing–pressing indentation method was used to evaluate the apparent ductility after modification. Figure 12 shows the morphology of the indentation track with a 200 N load and 10^4^ cycles. With influence of dynamic pressing–pressing, obvious radial cracks resulted at the corner of the track on the ZrN metallurgy layer and circumferential cracks resulted inside the tracks. This indicates that the initial ductility of the ZrN metallurgy layer was low. No obvious radial cracks and circumferential cracks with severe fatigue effect resulted with shot peening post treatment.

This indicates that the apparent ductility of the ZrN layer was improved by shot peening with the introduction of a reasonable residual compressive stress field to increase the cracking resistance of the ZrN metallurgy layer. In addition, the track dimensions on the ZrN+SP layer were smaller than those on the ZrN layer. This is attributed to strain hardening and a high residual compressive stress field on the compound modified layer. High ductility of the ZrN+SP compound layer is beneficial to improve its FW and FF behavior.

### 3.5. FW Behavior

**Figure 13:**
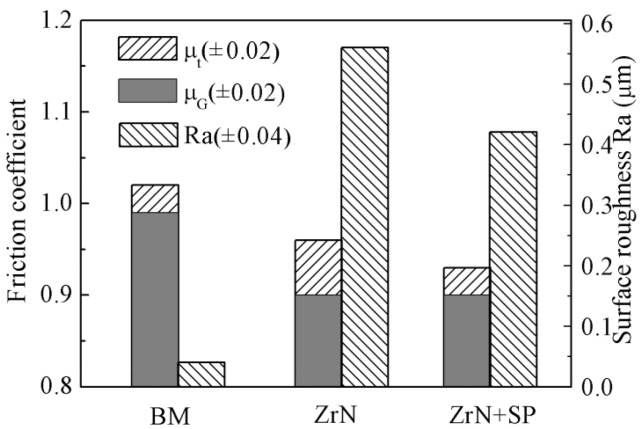
μ_t_, μ_G_ and the surface roughness (*R*a) of different modified specimens.

Modification to improve FF behavior should result in a good FW first. Research has indicated that the ZrN modification layer could increase the sliding wear resistance of titanium alloys [19]. However, most researches indicate that normal sliding wear and FW behavior is not equal. In the FW region, the contact mode is divided into adhesive, partial slipping and slipping modes according to the sliding amplitude and contact stress. The wear behavior of the slipping mode and normal sliding wear is in basic agreement. Using a displacement increment method, researchers have determined that the transition slipping amplitude between the partial slip and slip modes is 55 μm for a contact load *P* = 50 N/mm [22]. Figure 13 shows the average transition friction coefficient μ_t_ between the partial slip and slip modes, the average friction coefficient μ_G_ under slip mode and the surface roughness (*R*a) of the modified specimen. The value of μ_t_ is greater than that of μ_G_. This agrees with the fact that the static friction coefficient is generally higher than the dynamic friction coefficient. μ_t_ and μ_G_ of Ti6Al4V are nearly 1.0. μ_t_ and μ_G_ of the ZrN layer are smaller than that of Ti6Al4V because the ZrN layer can retard adhesive wear in the FW contact region. Compared with the ZrN layer, μ_t_ and μ_G_ of the ZrN+SP layer are both reduced slightly. This is because shot peening post treatment can smooth the ZrN layer to some extent or reduce the surface roughness (*R*a). Reducing the friction coefficient improves both the FW and FF behavior .

**Figure 14:**
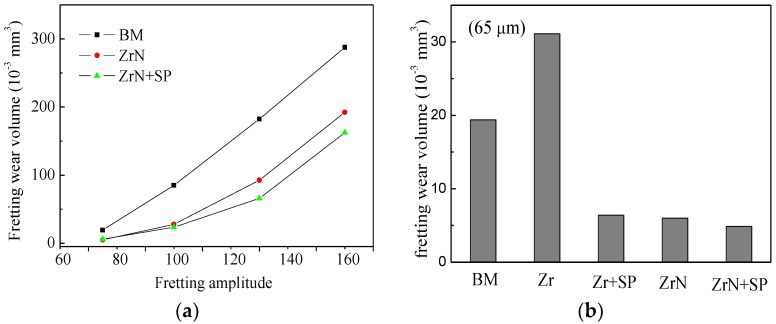
(**a**) Wear volume loss at different slip amplitudes and (**b**) comparison at 65-μm slip amplitudes.

Figure 14 shows the FW volume loss with different slipping amplitudes of Ti6Al4V base (BM), ZrN and ZrN+SP specimens. The FW volume loss increased with increasing FW slipping amplitude. This occurs because a higher slipping amplitude results in a greater FW distance. ZrN modification improved the FW resistance of Ti6Al4V alloy. This is attributed to two reasons. Firstly, the ZrN layer hardness is greater than that of the TiAl4V base. Secondly, the friction coefficient of ZrN is slightly lower than that of Ti6Al4V base, which therefore reduces the friction shear stress. Thirdly, ZrN modification improves antioxidant properties of titanium alloy. Shot peening post treatment improves the FW resistance of the ZrN layer further. A higher slipping amplitude results in a more obvious improvement because of strain hardening and the introduction of a surface residual stress field with high amplitude and depth, which is beneficial to improve fatigue wear behavior.

**Figure 15:**
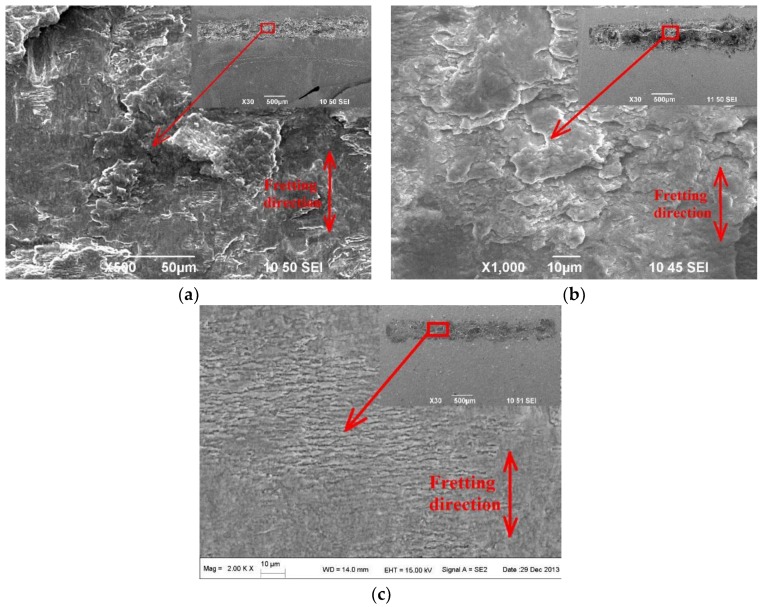
Morphology of FW tracks for different modified specimens. (**a**) Ti6Al4V alloy base; (**b**) ZrN; (**c**) ZrN+SP.

**Figure 16:**
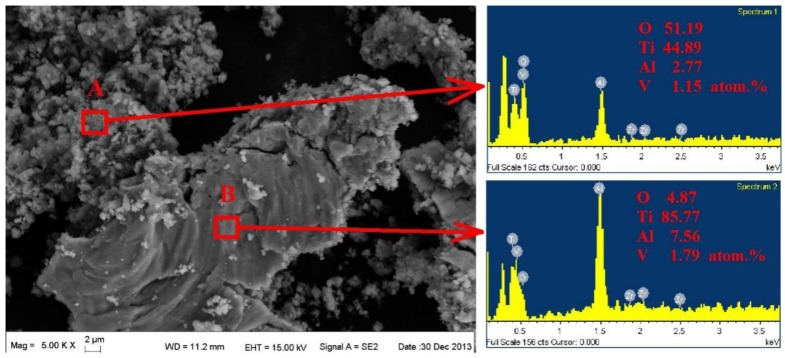
Pattern and energy-dispersive X-ray spectroscopy analysis of wear debris of Ti6Al4V alloy.

Figure 15 shows the morphology of the FW track on the BM, ZrN and ZrN+SP specimens. The test slipping amplitude was 65 μm and the FW life was 10^5^ cycles. The FW was severe, and delaminations existed in the fretting contact region of Ti6Al4V base. During FW, plastic deformation accumulates and dislocations increase. So, the surface plasticity decreases and the fragility increases to initiate microcracks in work hardening subsurface regions. Microcracks propagate to the surface and the subsurface breaks away from the base. Titanium alloy delaminations from base oxides rapidly with the effect of FW and then TiO_2_ oxidations with high hardness (Figure 16) accelerate abrasive wear in FW.

The degree of wear on the ZrN sample track was lower than that of Ti6Al4V base. Local delaminations occur, which indicates that fatigue wear is the main wear mechanism. This is because the ductility of the ZrN layer is low. The degree of wear of the compound-modified specimen (ZrN+SP) is lower than that of Ti6Al4V base or ZrN specimen. Shallow cracks exist in the normal direction of the slipping movement. This occurs because shot peening could introduce residual compressive stress and improve the apparent ductility to inhibit crack propagation in depth.

### 3.6. FF Behavior

**Figure 17:**
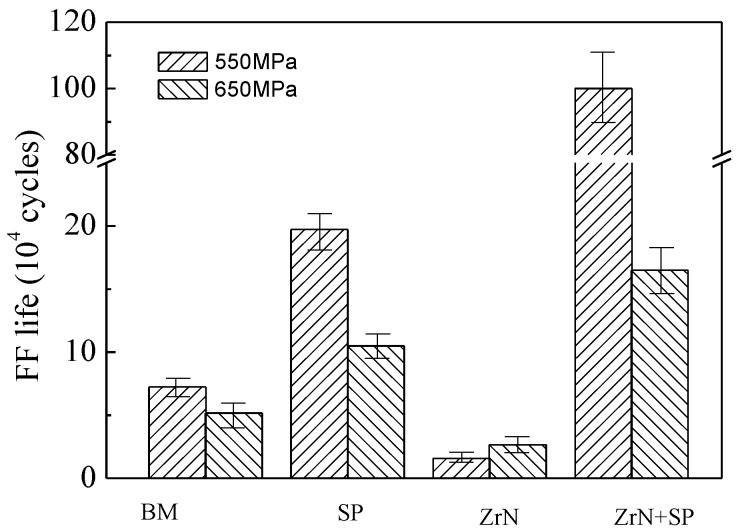
FF life of Ti6Al4V alloy with different modifications.

**Figure 18:**
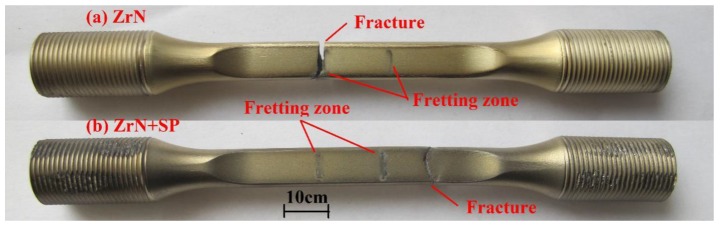
FF testing failure patterns of the ZrN and ZrN+SP specimens.

Figure 17 shows the FF lives of the BM, SP, ZrN and ZrN+SP specimens. For 550 MPa fatigue stress, shot peening improved the FF life of Ti6Al4V alloy by 1.7 times and ZrN modification reduced the FF life of Ti6Al4V alloy by 78%. ZrN+SP modification improved the FF life of Ti6Al4V alloy by 12.8 times, which is obviously more effective than SP alone. FF fractures of compound modified specimens do not lie in the fretting contact region, but at the edge corners outside the fretting contact region (shown in Figure 18). This fracture phenomenon of fracturing outside the fretting contact region has also been reported in the research [17]. When the maximum fatigue stress increased to 650 MPa, the FF life of the BM and ZrN specimens was reduced. The effectiveness to improve FF behavior for SP and ZrN+SP modification were both reduced visibly. Compared with that under 550 MPa maximum fatigue stress, the effectiveness for improving the FF life of the ZrN+SP specimens was reduced especially.

**Figure 19:**
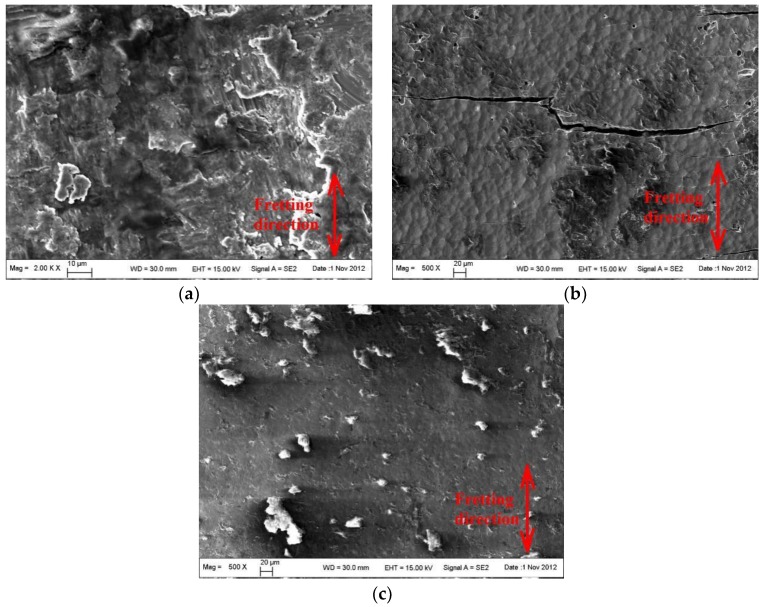
Fretting wear morphology of fretting fatigue specimens with different modifications after failure. (**a**) BM; (**b**) ZrN; (**c**) ZrN+SP.

Figure 19 shows the micromorphology in the fretting damage region of Ti6Al4V alloy specimens with different modifications after failure. Classical delaminations and fatigue cracks existed in the fretting contact region of Ti6Al4V alloy (Figure 19a). This is similar to the morphology of the FW region of the FW specimen. Local contact wear features existed and light wear resulted in the FW region of the ZrN specimen. Much brittle cracking existed, which indicates that crack initiation was easy. Local wear features existed and the degree of wear was light. Debris was transferred from the fretting pads and no obvious cracking occurred in the fretting contact region.

**Figure 20:**
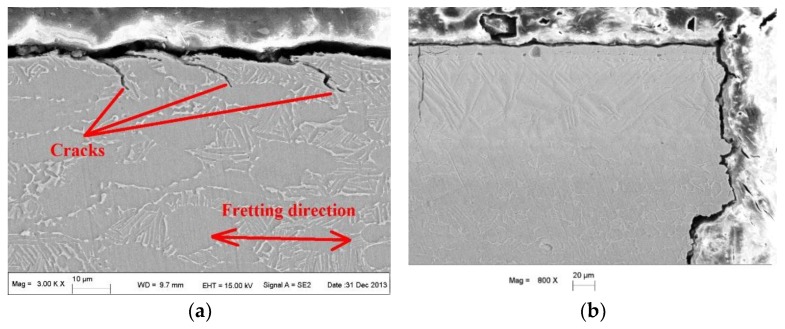

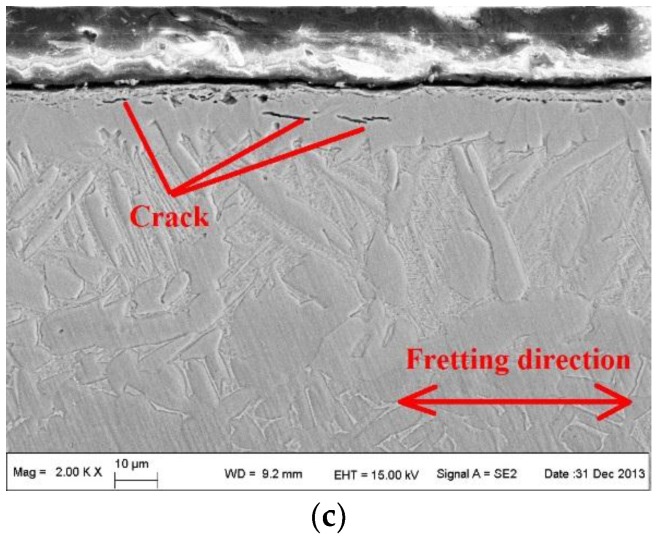
Cross-sectional micromorphology of FF specimen after failure with different modifications. (**a**) Ti6Al4V base; (**b**) ZrN; (**c**) ZrN+SP.

Figure 20 shows the micromorphology of the fretting damage contact region of Ti6Al4V FF specimens with different modifications. As can be seen from Figure 20a, during the initiation progress (I stage), cracks propagated in a bank angle with a specimen surface. As the crack length increased, the crack propagation direction tended to be normal to the extra alternating fatigue stress (that is, in a direction normal to the specimen surface). The test results indicate that the value of the bank angle and the depth of the crack are related to the material properties, fretting slipping status, contact stress, friction force, surface modification state, extra alternating stress status and so on [6,8,14]. As can be seen from Figure 20b, the FF crack of the ZrN specimen propagated normally to the specimen surface (or extra alternating fatigue force). No crack propagation existed in the first stage in the bank direction as titanium alloy. This occurs because it is difficult to form slip plastic deformation with effects of multi-axis stress in the fretting contact region, and fatigue cracks initiate and propagate depending on the effect of primary stress in the ZrN layer with high hardness and low ductility. It can also be seen from Figure 20b that secondary brittle fatigue cracks exist in the fretting contact region of the ZrN specimen. Its direction is the same as the normal direction of the extra alternating force. As can be seen from Figure 20c, although microcracks exist parallel to the ZrN+SP specimen surface, these cracks do not all develop to propagate in the depth direction of the FF specimen. Thus, fractures do not lie in the fretting contact region but in the edge corner with a higher stress concentration existing outside the fretting contact region. The failure mechanism of the ZrN+SP specimen is a plain fatigue fracture.

**Figure 21:**
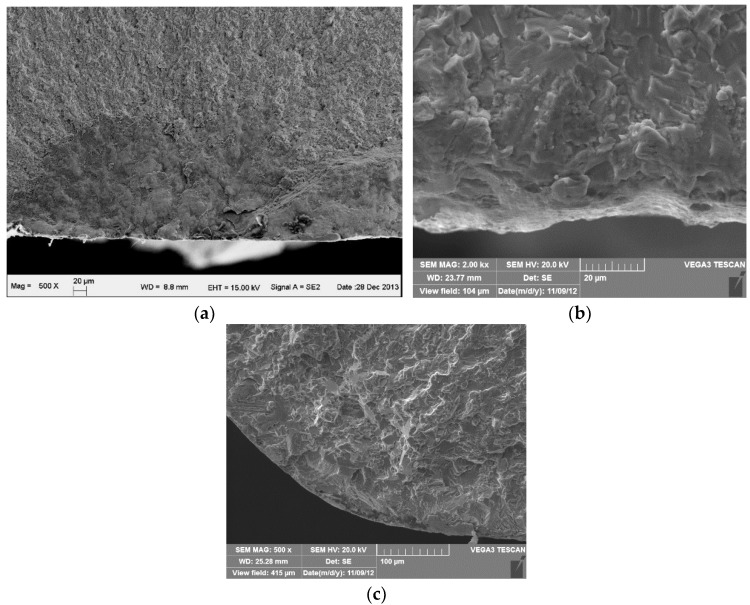
Fracture morphology of fatigue failure specimen with different modifications. (**a**) BM; (**b**) ZrN; (**c**) ZrN+SP.

Figure 21 shows the fracture morphology of FF or plain fatigue specimens with different modifications. The FF crack of the BM specimen initiates from a point source and propagates in the depth direction. Obvious abrasion marks are visible in the region of the crack source. The ZrN specimen exhibits features of a transgranular brittle cleavage fracture. This is related directly to the low ductility of the ZrN layer. With shot peening post treatment, the crack source of the ZrN specimen shifts from the FW region to the edge corner with high stress concentration outside the fretting contact region. That is, the fatigue resistance in the FW region is higher than that at the edge corner outside the FW region. The fatigue failure mechanism is transformed from FF to plain fatigue failure. This fracture transformation occurs because shot peening improves fatigue resistance in the FW region and inhibits crack initiation or propagation in the fretting contact region.

## 4. Discussion

The requirement to improve surface FW and FF resistances differs from a comparison of the results of FW and FF testing [4,5,6,7,8]. In this research, abrasive, oxidation, fatigue and adhesive wear exist (Figure 10 and Figure 11). When the slipping amplitude is small, fatigue and adhesive wear are the primary mechanisms. When the slipping amplitude is high, a large amount of abrasive and oxidation wear results. ZrN modification improves the surface hardness, load-bearing ability and anti-oxidation behavior and reduces the friction coefficient and adhesion inclination. So, ZrN modification could inhibit abrasive, oxidation, fatigue and adhesive wear to a certain extent, especially for abrasive and anti-oxidation wear. Therefore, with an increase in slipping amplitude, the surface of the ZrN alloying modification become more effective to improve FW resistance (Figure 14). When the ZrN layer is shot peened, a residual compressive stress can be introduced and the apparent ductility can be improved to increase the fatigue resistance and fatigue wear resistance of the ZrN layer [16,21]. Thus, the FW resistance of the ZrN could be improved further by shot peening.

However, the fatigue mechanism is the primary factor for FF failure. Improvement of the fatigue resistance is the first solution to improve the FF resistance of the titanium alloy. However, ZrN is the main phase on the superficial ZrN layer and its hardness is high and its ductility is low. The fatigue resistance of the ZrN phase is so low that fatigue cracks easily initiate rapidly and propagate with multi-axis stress in the contact region. Therefore, the FF resistance of the ZrN specimen is lower than that of the BM specimen. Shot peening causes superficial layer work hardening and introduces surface residual compressive stress. The crack-closing force is increased and the friction coefficient is reduced. Shear stress in the contact region is reduced to inhibit fatigue crack initiation and early propagation to improve FF behavior of the titanium alloy [13,17]. Shot peening could introduce beneficial residual compressive stresses, reduce surface roughness and increase the subsurface hardness to improve its apparent ductility. The ZrN layer strength is high, the subsurface Zr–Ti solid solution layer has excellent work hardening and the amplitude and depth of the residual stress introduced by shot peening is high (Figure 11). Thus, the robust performance and anti-fatigue behavior of the ZrN+SP specimen is excellent. In addition, the ZrN specimen exhibits good FW resistance. Thus, shot peening could improve the FF resistance of the ZrN specimen visibly. The FF resistance of the ZrN+SP specimen is not only higher than that of the titanium alloy, but also than that of the shot peening specimen alone. The FF resistance of the compound-modified specimen is higher than the plain fatigue resistance of the edge corner with great stress concentration, which results in fractures occurring in the specimen’s edge corner outside the fretting damage area. As can be seen from Figure 17, shot peening and ZrN+SP compound-treated specimens improve the FF resistance of the titanium alloy specimen with the maximum fatigue stress increasing from 550 to 650 MPa. This occurs because the effectiveness for improving the FF resistance of the titanium alloy is reduced with the extra fatigue force increasing to make the residual stress decay faster in fatigue testing.

Because FW and the extra fatigue force combine to accelerate FF damage, coordination of shot peening and surface modification may improve FW and fatigue resistance behavior and improve the FF property of the titanium alloy. However, inappropriate compound treatment does not improve the FF resistance of titanium alloy. For example, ion implanting of N and C, plasma spraying of CuNiIn and plasma deposition of a TiN film combined with shot peening improves FF resistance less than shot peening alone [3,14]. This is because the adhesive strength of samples after these modifications is too low for them to endure shot peening treatment. The only suitable combined treatment would be to conduct shot peening first and then conduct surface spraying or deposition. The residual compressive stress introduced by shot peening is relaxed under the evaluated temperature and defects introduced by shot peening harm the surface integrity of the above modifications. Thus, improvements in FF behavior with combination treatment are reduced. In this research, a ZrN alloying layer was prepared using the plasma metallurgy technique. Adhesion between a titanium base and the modified layer was conducted metallurgically. Thus, the adhesive strength of the ZrN layer could endure shot peening post treatment. Therefore, prior ZrN treatment would not reduce the FF resistance of shot peening, but would increase the capacity of shot peening to improve the titanium alloy FF resistance.

## 5. Conclusions

(1) A ZrN metallurgy layer with an 8-μm-thick ZrN phase superficial layer was prepared on Ti6Al4V alloy using glow plasma metallurgy. The subsurface was a Zr–Ti solid solution layer with a certain nitrogen element. The total modified layer was 60 μm thick. The surface hardness was 1775 HK_0.1_ and changed gradually with an increase in depth.

(2) A ZrN modified layer improved the FW resistance of Ti6Al4V alloy. A greater slipping amplitude resulted in an increase in the extent of FW resistance. Shot peening post treatment could improve the FW resistance of the ZrN layer on Ti6Al4V alloy further.

(3) A ZrN metallurgy layer reduced the FF resistance of Ti6Al4V alloy, which is different from its influence on FW behavior of the titanium alloy. This is because the surface hardness of the ZrN metallurgy layer is high and its ductility is low with a high notch sensitivity and therefore, its fatigue resistance is poor. The ZrN+SP combined treatment clearly improved the FF resistance. It was more effective than that of shot peening alone. This can be attributed to its good robust performance of FW resistance and fatigue resistance, which together improve FF resistance.
